# Highly localized divergence within supergenes in Atlantic cod (*Gadus morhua*) within the Gulf of Maine

**DOI:** 10.1186/s12864-017-3660-3

**Published:** 2017-03-31

**Authors:** Bryan T. Barney, Christiane Munkholm, David R. Walt, Stephen R. Palumbi

**Affiliations:** 1 Department of Biology, Hopkins Marine Station, Stanford University, 120 Ocean View Boulevard, Pacific Grove, CA 92950 USA; 2 Gloucester Marine Genomics Institute, 55 Blackburn Center, Gloucester, MA 01930 USA

**Keywords:** Atlantic cod, *Gadus morhua*, Linkage disequilibrium, Linkage map, Genomic divergence, Supergene, Gulf of Maine

## Abstract

**Background:**

Atlantic cod (*Gadus morhua*), is known to vary genetically across the North Atlantic, Greenland, and Newfoundland. This genetic variation occurs both spatially and temporally through decades of heavy fishing, and is concentrated in three linkage disequilibrium blocks, previously defined by pedigreed linkage mapping analysis. Variation within these genomic regions is correlated with both seawater temperature and behavioral ecotype. The full extent and nature of these linkage groups is important information for interpreting cod genetic structure as a tool for future fisheries management.

**Results:**

We conducted whole genome sequencing for 31 individual cod from three sub-populations in the Gulf of Maine. Across the genome, we found 3,390,654 intermediate to high frequency Single Nucleotide Polymorphisms (SNPs). We show that pairwise linkage analysis among these SNPs is a powerful tool to detect linkage disequilibrium clusters by recovering the three previously detected linkage groups and identifying the 1031 genes contained therein. Across these genes, we found significant population differentiation among spawning groups in the Gulf of Maine and between Georges Bank and Gulf of Maine. Coordinated divergence among these genes and their differentiation at both short and long spatial scales suggests that they are acting as linked supergenes in local adaptation of cod populations.

**Conclusions:**

Differentiation between SNPs in linkage disequilibrium blocks is the major signal of genetic differentiation between all groups tested within the Gulf of Maine. Our data provide a map of genes contained in these blocks, allowing an enhanced search for neutral genetic structure for demographic inference and fisheries modeling. Patterns of selection and the history of populations may be possible to identify in cod using this description of linkage disequilibrium blocks and future data sets to robustly separate neutral and selected genetic markers.

**Electronic supplementary material:**

The online version of this article (doi:10.1186/s12864-017-3660-3) contains supplementary material, which is available to authorized users.

## Background

Genomic islands of divergence are the result of selection on regions within the genome undergoing adaptive divergence between subgroups within a species [1, 2]. These islands can manifest as regions of strong linkage disequilibrium due to genetic hitchhiking, while other regions within the genome are recombined through gene flow [1, 3, 4]. Adaptive divergence in regions of the genome may be an important driving force in speciation, particularly when they create differences in critical biological processes such as reproductive behavior and timing [1, 5], although the conditions under which these processes can occur may be limited [6, 7]. A significant body of literature has been published on speciation with gene flow [8–11], and islands of divergence are often cited as a possible explanation of this phenomenon.

Within species, islands of divergence are associated with differential selection across variable environments. In the threespine stickleback within Lake Constance in Germany, for example, strong differentiation between migratory lake and resident stream ecotypes is associated with differentiation across a set of strongly linked genes [12]. Chromosomal inversions often lead to large regions of strong linkage disequilibrium [13], and studies of these inversion polymorphisms in various *Drosophila* species have shown that haplotype frequencies of these inversions change along environmental clines. For example, the In(3R)Payne inversion in *D. melanogaster* quickly evolved different frequencies across latitudes in Australia. Moreover, allele frequencies of the more equatorial haplotype of the inversion polymorphism have increased across the entire cline, suggesting a population adaptive response to increased temperature over 30 years [14–17]. A strikingly similar pattern occurs in D. *subobscura*, where the more southerly haplotype of the O inversion has increased in frequency over the last 15 to 30 years in all European populations tested [18]. In the yellow monkey flower (*Mimulus guttatus*), populations show differential fitness across coastal and inland habitats based on a set of genes linked within an inversion [19]. All in all, ‘supergenes’ are receiving more attention as a combination of genomic information and evolutionary theory shows how multi-locus selection and linkage can evolve to generate local adaptation [20–22].

The relationship between environmental conditions and genomic islands has also been studied in the Atlantic cod, *Gadus morhua*. Borza et al. (2010) and Hubert et al. (2010) developed a panel of ~1500 SNPs derived from expressed sequence tags (EST) and used them to create linkage maps from pedigreed cod populations [23, 24]. They discovered three genomic islands of divergence that were highly associated with mean ocean temperatures across the northern Atlantic [25, 26]. The same set of markers also describes ecotype divergence between migratory and stationary cod populations in Iceland and Norway [27], as well as small scale spatio-temporal patterns of population structure in Newfoundland [28, 29]. Using a custom SNP array from the recently published improved assembly of the cod genome [30], genomic differences were described between oceanic and coastal populations in the North Sea [31] and the Norwegian Sea [32]. In all these cases, genetic differences among populations at both oceanic and local scales are thought to be driven by environmental selection mediated by a suite of genes in several linkage groups, not by demographic barriers to gene flow. At smaller geographic and temporal scales, population genetic analyses of cod in the Gulf of Maine have focused on a suite of microsatellite and protein markers in a large number of individuals across many populations [33]. Most of the significant differences compiled in Zemeckis et al. [33] are from individual loci which appear to be under selection, rather than the result of neutral genetic structure between populations [34, 35].

To date, the loci within the cod linkage groups have been identified through breeding studies and classic quantitative trait loci (QTL) approaches. Moreover, the search for neutral genetic differentiation, outside the known linkage groups, has been based on relatively few loci compared to genome-level data sets. Both the searches for selected genes and those that might reflect neutral genetic differentiation have not yet taken advantage of full-genome approaches [36]. Here, we use high throughput sequencing and population genomic analyses to examine the degree and spatio-temporal distribution of linkage disequilibrium clusters throughout the genome of Atlantic cod in the Gulf of Maine and Georges Bank. We then use this information to eliminate linked genes from the analysis and use the remaining genes to test for patterns of neutral genetic differentiation.

Using complete genome sequencing, we describe a panel of 3,390,654 single nucleotide polymorphisms (SNPs) throughout the cod genome. Pairwise linkage analysis shows SNPs within 1031 genes falling into three cod ‘supergenes’, two of which show population divergence within the Gulf of Maine. Genes within these linkage blocks include DNA structural proteins and chromatin assembly genes, metabolic and catabolic genes, meiosis regulation and oocyte maturation genes, odorant receptors, egg coat structural proteins, heat shock proteins and many cell signaling genes that might be involved in environmental adaptation, habitat choice or mating. Once linkage disequilibrium blocks were identified and excluded from the analysis, our limited population data show no signs of neutral genetic differentiation in the three populations we sampled. Though our power to detect neutral structure is low with such few samples, comparison of many SNPs suggests that the signature of population differentiation is largely driven by shifts in the supergenes, possibly driven by present or past patterns of natural selection.

## Methods

### Sample collection

**Fig. 1:**
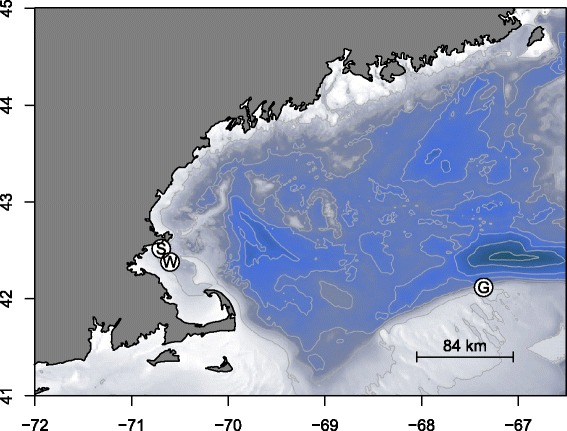
Sampling locations used in this study. Twenty one adults in spawning condition were sampled in northwestern Massachusetts Bay within the Gulf of Maine. These were subdivided into 11 spring spawning cod (site S) and 10 winter spawning cod (site W). Ten adults in spawning condition were sampled from Georges Bank (site G). Figure was generated using the *marmap* package in R

Fin-clip samples from adult Atlantic cod in spawning condition were collected and stored frozen prior to preparation of DNA libraries. A total of 31 individuals were sampled: 10 from Georges Bank, and 21 from the Gulf of Maine. Within the Gulf of Maine, the sample was subdivided into two groups, based on spawning season: 10 cod were sampled from a winter spawning group, and 11 cod from a spring spawning group (Fig. 1). These samples were taken from cod stocks in the northwestern part of Massachusetts Bay near Cape Ann, as studies of cod sampled from this region showed evidence of temporal population substructure based on breeding season [34, 35]. Samples were collected in March 2014 for the Georges Bank population, and in December 2013 and June 2014 for the Gulf of Maine winter and spring populations, respectively (Table 1).

**Table 1:** Cod sampling summary

Stock	Date	Samples (n)	Latitude	Longitude
Gulf of Maine winter	Dec 2013	10	42.3813	-70.6016
Gulf of Maine spring	June 2014	11	42.5177	-70.6921
Georges Bank	Mar 2014	10	42.1192	-67.3575

Locations, dates, and number of samples collected at each sampling site

### DNA extraction, library preparation, sequencing

Genomic DNA (gDNA) from each fin clip was prepared by digesting entire fin clips (approximately 1 cm^2^) in 500 μL of Digestion Mixture (1 μg/μL Proteinase K, 50 mM EDTA, 5% Tween 20, 0.5% Triton-X 100, 800 mM GuHCl, 0.5% SDS) at 50 °C for 1 h. After digestion, the samples were centrifuged at 13,000 rpm for 10 min; the supernatant was recovered, and then mixed with 50 μL of 3 M potassium chloride to precipitate the SDS. An equal volume of isopropyl alcohol was added to precipitate nucleic acids, and the pellet was washed with 70% ethanol. DNA/RNA was resuspended in 100 μL of 10 mM Tris-HCl pH 8.0 and treated with 5 μL of 10 mg/mL RNase A. Another isopropyl alcohol precipitation was performed, then genomic DNA was isolated using AMPure XP beads. Total gDNA was resuspended and stored in TE buffer (10 mM Tris-HCl, 1 mM EDTA, pH 8).

Sequencing libraries were prepared from 50 ng of template DNA using the Illumina Nextera DNA library preparation kit (Illumina, San Diego, CA) following their standard protocol. Samples were individually barcoded to allow for multiplexing during sequencing. Each library was independently size selected using Ampure XP beads (0.6 volume beads to 1.0 volume DNA) and checked for adequate sizing via gel electrophoresis. Libraries were quantified by qPCR (KAPA Illumina Quantification Kit), then normalized and mixed in equimolar fashion.

One set of samples, consisting of five individuals from each sampled location, was sequenced on an Illumina HiSeq 2000 using 100 base paired end reads. Each batch of five samples from the original sample groupings were mixed and multiplexed across a distinct eight-lane HiSeq flowcell, resulting in approximately 80x coverage across the genome (predicted size ~800 Mb). The remaining samples (five winter, six spring, and five Georges Bank) were pooled and sequenced to less depth of coverage (~25x) on three runs of an Illumina NextSeq 500 using 150 base paired end reads.

### Read mapping and variant calling

Raw reads were trimmed of any residual adapter sequences, and low-quality base calls were removed using a baseline Phred score of 20 or less. Individual reads were retained if the length was > 70 bases, and reads were re-paired using Trimmomatic [37]. Read pairs were then mapped to the reference genome file gadMor2.fasta [30] using bowtie2, with end-to-end alignment, 22 base seed length, two re-seed attempts with no mismatches allowed in the seed, a seed interval of 17 bases, and all other settings at default values. Reads that became unpaired due to quality or trimming issues were mapped separately using the same parameters. The resulting mapped files were sorted and indexed using samtools [38] and duplicated reads were removed using Picard tools (http://broadinstitute.github.io/picard) for downstream SNP discovery and analysis.

SNP discovery was performed on individual samples using FreeBayes version 0.9.10-3-g47a713e, using the default settings [39]. Variants were filtered using vcftools to only include biallelic SNPs with a minimum minor allele frequency of 0.05 using vcftools version 0.1.12b [40]. Filtering by minor allele frequency ensures that SNP loci with extremely low diversity are removed from downstream analysis, as they are essentially uninformative. The resultant SNP list was further limited to those SNPs that were genotyped (Q > 30) in all 31 samples.

### Data analysis

We limited our analysis of linkage disequilibrium to linkage groups (hereafter LG) 2, 7, and 12, as these regions contain the three “islands of divergence” studied in this species. Within each LG, we extracted every 250th SNP, and performed pairwise linkage disequilibrium analysis using the LD function in the ‘genetics’ package (version 1.3.8.1) in R version 3.03 (R development core team 2015). We determined the correlation coefficient (as r) between the paired genotypes of all 31 individuals, and reported the *r* ^2^ value to remove any arbitrary sign introduced by intermediate calculations. To examine the genomic extent of linkage disequilibrium effects within each LG, mean *r* ^2^ was calculated for every 250th SNP within each LG, as was pairwise F_ST_. The edges of each linkage disequilibrium block (hereafter LD block) were located by finding the regions with high mean LD (mean *r* ^2^ > 0.1) and determining the first and last SNP with similar mean *r* ^2^ values to the block. As there are 250 SNPs between the first high LD SNP and its subset neighbor and untested linked loci are located within this space, we placed the LD block edges on the SNPs adjacent to the ones with the first and last high LD value.

Using the gene model information from the genome, we determined which genes were in each LD block, and performed enrichment tests of Gene Ontology (GO) categories of the genes within the resultant LD block using the weight01 algorithm within the ‘topGO’ package version 2.24.0 [41] in R. Additionally, all SNPs throughout the genome were binned into one of three classes; exon, intron, or intergenic. We determined the proportion of SNPs within each class inside each LD block and throughout the genome, to test if SNPs of any class were enriched within LD blocks.

To test for population differentiation, we filtered all SNPs for those found within exons and created subsets for analyses. For exonic SNPs on linkage groups 2, 7, and 12 we used all available exonic SNPs, each partitioned into two groups: those within the LD block and those outside of the block. For the remainder of the genome, we created a subset of every 10th exonic SNP. We focused solely on SNPs within exons for this analysis, as we are interested in both neutral population structure and adaptive divergence between spawning sites/times, and SNPs within the coding regions of genes can be used to examine both processes. From this subset of SNPs, Weir and Cockerham’s pairwise F_ST_ was calculated among the three populations using the wc function from the ‘hierfstat’ package [42, 43] within R. This was performed for exonic SNPs within each LD block, SNPs on the same linkage group but not in the LD block, and all exonic SNPs not on linkage groups 2, 7, or 12. Principal component analysis (PCA) was performed on SNPs both within and outside of each LD block using the ‘prcomp’ function within the standard release package of R.

To test for neutral population structure, we utilized the same subsets of exonic SNPs used for F_ST_ and PCA analysis above. To avoid potential confounding selection with population structure, SNPs within the linkage blocks on LG02, LG07, and LG12 were removed from this dataset and tested separately. With these sets of SNPs, we tested for population structure using the admixture algorithm NGSadmix [44]. For each admixture analysis, we tested the model against a number of subpopulation divisions (k = 2 to k = 5), and chose the k value that had the greatest likelihood among all k values tested using the Evanno method [45].

## Results

### Linkage disequilibrium block determination

**Fig. 2:**
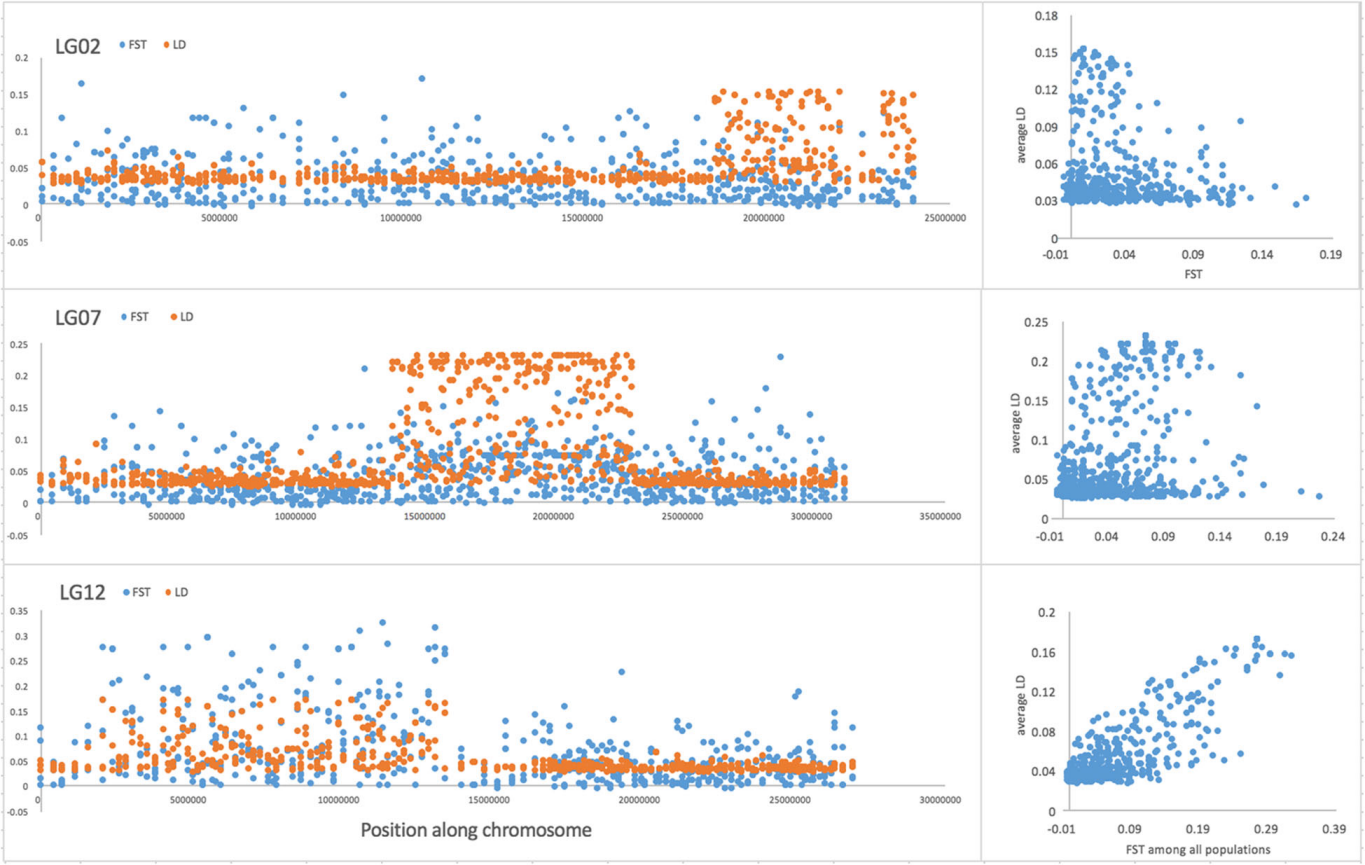
Linkage and population differentiation for chromosomes 2, 7 and 12 inside and outside linkage blocks. Mean pairwise LD (as *r* ^2^) and F_ST_ are shown on the y-axis for every 250th SNP, with SNP position within its linkage group along the x-axis. The right hand figures show a regression of LD vs F_ST_ for each SNP

After mapping reads to the genome assembly, we identified 3,390,654 biallelic SNP variants with a minor allele frequency greater than 0.05 that were confidently genotyped in all 31 samples. There were 147,356 SNPs located on LG02, 204,124 SNPs on LG07, and 139,785 SNPs on LG12. To find the edges of the LD blocks, pairwise linkage disequilibrium (as r^2^) and F_ST_ was calculated for every 250th SNP in LG02 (589 SNPs), LG07 (816 SNPs), and LG12 (559 SNPs). From these data, mean pairwise *r* ^2^ values were determined for each SNP (Fig. 2). Each LD block within a linkage group shows a highly contiguous and sharply defined region of elevated mean linkage disequilibrium (mean *r* ^2^ > 0.1) in comparison with the surrounding SNPs (mean *r* ^2^ ~ 0.03 to 0.04). F_ST_ was less useful in determining the edges of LD blocks, as this varied over wide ranges on SNPs within linkage groups 2 and 7. Interestingly, F_ST_ and LD appear correlated in linkage group 12 (Fig. 2, bottom right), perhaps due both to the large extent of the linkage region and the high degree of differentiation seen in this sample set between the winter spawning cod and Georges Bank populations in the linked loci.

To capture potentially linked loci between the subset SNP loci, the starting base for each LD block was identified as the SNP immediately before the first SNP with a mean *r* ^2^ > 0.1 in the LD block, and the ending base was the first SNP after the last base with a mean *r* ^2^ > 0.1 in the LD block, resulting in LD blocks of 5.6 Mb (LG02), 9.3 Mb (LG07) and 11.6 Mb(LG12) in length (Table 2). There was a linkage gap of about 2 million bases in LG02, inside which SNPs were not linked to one another or to the SNPs in the rest of the linkage group (Fig. 2, top left). For LG02, 58 of the 174 SNPs within the LD block (~33%) were strongly linked (mean pairwise *r* ^2^ > 0.1), while 170 of the 312 SNPs (~54%) in LG07 and 42 of the 246 SNPs (~17%) in LG12 were strongly linked. Our findings agree with regions of linkage disequilibrium identified previously by EST-derived SNPs [23, 25–27] and Illumina SNP arrays [31, 32]. However, our analysis provides a higher degree of resolution of both the range and edges of these LD blocks, as previous work has described LD blocks in terms of relative centimorgans(cM) rather than actual base positions.

**Table 2:** Linkage disequilibrium block extent, and approximate length of each block

	Starting base	Ending base	# genes	length
LG02	18,442,392	24,053,701	294	5.6 Mb
LG07	13,743,341	23,039,143	306	9.3 Mb
LG21	2,039,256	13,611,225	437	11.6 Mb

### Genetic content of LD blocks

Using the available gene models from the cod genome and the start and stop base positions for each LD block described above, we determined which genes were within each LD block. Of the 1095 genes on LG02, 294 were found within the boundaries of the LD block. GO enrichment analysis on genes within this LD block showed several overlapping GO terms with significant enrichment (*p*-*adj* < 0.01 after false-discovery rate correction) centered on genes involved with DNA/chromatin structuring (Table 3). Genes within the significant GO terms consisted primarily of histone proteins involved in the assembly of the nucleosome: 14 of the 27 genes in the significant GO terms coded for histone proteins (Table 4). Additionally, two histone modification genes, histone-lysine N-methyltransferase and histone deacetylase are also located within this linkage block.

**Table 3:** GO enrichment analysis of linkage disequilibrium block on LG02

GO.ID	Term	Annotated	Significant	Fisher pval	padj
GO:0006325	chromatin organization	142	18	1.70E-14	**2.52E**-**11**
GO:0065004	protein-DNA complex assembly	105	16	2.80E-14	**2.52E**-**11**
GO:0071824	protein-DNA complex subunit organization	105	16	2.80E-14	**2.52E**-**11**
GO:0051276	chromosome organization	178	19	7.10E-14	**4.76E**-**11**
GO:0006334	nucleosome assembly	100	15	2.50E-13	**9.39E**-**11**
GO:0031497	chromatin assembly	100	15	2.50E-13	**9.39E**-**11**
GO:0034728	nucleosome organization	100	15	2.50E-13	**9.39E**-**11**
GO:0006333	chromatin assembly or disassembly	101	15	2.90E-13	**9.57E**-**11**
GO:0006323	DNA packaging	106	15	6.00E-13	**1.77E**-**10**
GO:0071103	DNA conformation change	112	15	1.40E-12	**3.65E**-**10**
GO:0034622	cellular macromolecular complex assembly	180	16	1.30E-10	**3.26E**-**08**
GO:0043933	macromolecular complex subunit organization	324	20	3.90E-10	**8.64E**-**08**
GO:0006461	protein complex assembly	244	17	1.50E-09	**2.91E**-**07**
GO:0070271	protein complex biogenesis	244	17	1.50E-09	**2.91E**-**07**
GO:0065003	macromolecular complex assembly	258	17	3.60E-09	**6.38E**-**07**
GO:0071822	protein complex subunit organization	271	17	7.50E-09	**1.24E**-**06**
GO:0022607	cellular component assembly	308	18	7.90E-09	**1.24E**-**06**
GO:0006996	organelle organization	432	21	1.00E-08	**1.50E**-**06**
GO:0044085	cellular component biogenesis	339	18	3.50E-08	**4.95E**-**06**
GO:0016043	cellular component organization	636	22	1.70E-06	**0.00023**
GO:0071840	cellular component organization or biogenesis	666	22	3.70E-06	**0.00047**
GO:0042157	lipoprotein metabolic process	32	5	2.70E-05	**0.0032**
GO:0044711	single-organism biosynthetic-process	295	9	0.0061	0.7060
GO:0006897	endocytosis	35	3	0.0071	0.7895
GO:0043543	protein acylation	12	2	0.0078	0.8318
GO:0016568	chromatin modification	38	3	0.0089	0.8645

Using topGO, (in R), enrichment analysis shows multiple GO categories that may be enriched in the LD block from LG02, mostly centered on DNA/chromatin structuring. Total number of annotated genes in each GO category is shown, as well as those within the LD block that are significant (Fisher test *p* < 0.01). Multiple-test correction (calculated as false-discovery rate, ‘fdr’) is shown in *p*-*adj* column, *p*-values < 0.05 shown in bold

**Table 4:** Genes in significant GO terms within LD block on LG02

Gene	Starting base	Gene description
GAMO_00059458	18475327	zgc:112234: Histone H2B 1/2 (Danio rerio)
GAMO_00059459	18476591	Histone H2A (Oncorhynchus mykiss)
GAMO_00059461	18489582	TGas006m08.1: Histone H4 (Xenopus tropicalis)
GAMO_00059462	18491420	Histone H2A (Oncorhynchus mykiss)
GAMO_00059465	18506398	Histone H2A (Oncorhynchus mykiss)
GAMO_00059556	19188916	STRA13: Centromere protein X (Homo sapiens)
GAMO_00059593	19396062	Crebbp: CREB-binding protein (Rattus norvegicus)
GAMO_00059696	20010135	NMT1: Glycylpeptide N-tetradecanoyltransferase 1 (Homo sapiens)
GAMO_00059920	21260662	Coro1b: Coronin-1B (Mus musculus)
GAMO_00059924	21287842	suv420h2: Histone-lysine N-methyltransferase SUV420H2 (Xenopus laevis)
GAMO_00059998	21707418	KCNA1: Potassium voltage-gated channel subfamily A member 1 (Homo sapiens)
GAMO_00060305	23130440	tmem11: Transmembrane protein 11%2C mitochondrial (Danio rerio)
GAMO_00060314	23170545	Histone H2A (Oncorhynchus mykiss)
GAMO_00060318	23177224	Histone H2A (Oncorhynchus mykiss)
GAMO_00060319	23198635	Histone H3 (Urechis caupo)
GAMO_00060320	23199855	Histone H2A (Oncorhynchus mykiss)
GAMO_00060321	23201514	TGas006m08.1: Histone H4 (Xenopus tropicalis)
GAMO_00060322	23202645	Histone H3 (Urechis caupo)
GAMO_00060324	23204017	Histone H2B (Salmo trutta)
GAMO_00060364	23359510	Pigq: Phosphatidylinositol N-acetylglucosaminyltransferase subunit Q (Mus musculus)
GAMO_00060373	23393752	Histone H2A (Oncorhynchus mykiss)
GAMO_00060375	23400842	TGas006m08.1: Histone H4 (Xenopus tropicalis)
GAMO_00060376	23401993	Histone H3 (Urechis caupo)
GAMO_00060390	23480761	HDAC9: Histone deacetylase 9 (Homo sapiens)
GAMO_00060462	23855114	APOL3: Apolipoprotein L3 (Homo sapiens)
GAMO_00060465	23866729	APOL3: Apolipoprotein L3 (Homo sapiens)
GAMO_00060467	23891523	APOL3: Apolipoprotein L3 (Homo sapiens)

While several GO terms were significantly enriched, many of them collapsed to the same set of DNA structural genes, including several genes encoding all four major histone proteins that make up the nucleosome

Within the 1166 genes on LG07, 306 were located within its LD block, including genes involved with signaling and metabolic processes, though no GO categories remained significant after false-discovery rate correction (Table 5). Similarly, the LD block within LG12 contained 437 genes out of the 975 genes in that linkage group, and no significant enrichment of any GO categories remained after false-discovery rate correction (Table 6). Specific genic content within each block can be found in the Additional files 1, 2, 3 for this publication.

**Table 5:** GO enrichment analysis of linkage disequilibrium block on LG07

GO.ID	Term	Annotated	Significant	Fisher pval	p-adj (fdr)
GO:0006855	drug transmembrane transport	5	2	0.0014	1
GO:0006066	alcohol metabolic process	24	3	0.0028	1
GO:0015893	drug transport	8	2	0.0038	1
GO:0042493	response to drug	8	2	0.0038	1
GO:0044765	single-organism transport	1098	23	0.0044	1
GO:0023051	regulation of signalling	275	9	0.0054	1
GO:1902578	single-organism localization	1118	23	0.0055	1
GO:1901615	organic hydroxy compound metabolic process	31	3	0.0058	1
GO:0006071	glycerol metabolic process	10	2	0.0059	1
GO:0019400	alditol metabolic process	10	2	0.0059	1

Using topGO, (in R), enrichment analysis shows multiple GO categories that may be enriched in the LD block from LG07, including genes involved with signal transport and metabolic processes. Total number of annotated genes in each GO category is shown, as well as those within the LD block that are significant (Fisher test *p* < 0.01). Multiple-test correction (calculated as false-discovery rate, ‘fdr’) is shown in *p*-*adj* column

**Table 6:** GO enrichment analysis of linkage disequilibrium block on LG12

GO.ID	Term	Annotated	Significant	Fisher pval	p-adj (fdr)
GO:0051648	vesicle localization	4	2	0.002	1
GO:0051650	establishment of vesicle localization	4	2	0.002	1
GO:0009057	macromolecule catabolic process	144	8	0.0051	1
GO:0051656	establishment of organelle localization	7	2	0.0067	1
GO:0051640	organelle localization	8	2	0.0088	1

Using topGO, (in R), enrichment analysis shows multiple GO categories that may be enriched in the LD block from LG12. Total number of annotated genes in each GO category is shown, as well as those within the LD block that are significant (Fisher test *p* < 0.01). Multiple-test correction (calculated as false-discovery rate ‘fdr’) is shown in *p*-*adj* column

In addition to the genic content within each LD block, we evaluated the distributions of SNP locations (classified as intergenic, exon, or intron) of all SNPs located on linkage groups 1 through 23 (*n* = 3,283,653 SNPs). This number is slightly smaller than the total number of SNPs passing our filters (*n* = 3,390,654) due to unused SNPs located on the few remaining unscaffolded contigs as well as mitochondrial DNA, yet still represents 97% of all identified SNPs. Proportions of each SNP type were calculated within each LD block, as well as the remaining SNPs not on linkage groups 2, 7, or 12 (Table 6). SNP density (as # SNPs/base) was also determined within each LD block and in the remainder of the genome as above. For the LD block on LG02, there was both an increase in overall SNP density (from 5.66e-03 to 7.72e-03, a 36% increase) and an increase in the proportion of exonic SNPs (from 0.065 to 0.089, a 37% increase) compared to genome-wide values. We also found a 50% increase over the genome-wide SNP density within the LD block on LG07 (from 5.66e-03 to 8.36e-03 SNPs/base), though there were no significant changes in the proportions of SNP type within this group and no deviations from expected polymorphism rates in LG12 (Table 7).

**Table 7:** Comparison of genome contents and population divergence of cod chromosome linkage groups 2, 7 and 12

Group	# SNPs	Block length (Mb)	SNP linked	# genes	Genes in LD	SNP density	proportion exonic SNPs	Fst in LD block	FST vs LD	FST outside LD block	Notes
LG02	43,341	5.6	33	1095	294	7.72E-03	0.089	0.098	negative	0.0025	discontinuous block
LG07	77,751	9.3	54	1166	306	8.36E-03	0.064	0.089	positive	0.0004	
LG12	61,251	11.6	17	975	437	5.29E-03	0.071	0.173	higly positive	0.0022	marked lack of SNPs adjacent to linkage block
Not 2, 7, or 12	2,792,388	493.2									

### Differentiation among sampled populations

The subsets of exonic SNPs used for population structure analyses contained a total of 54,030 SNPs. F_ST_ analysis of linkage group 2 used 11,241 SNPs (3874 in LD block, 7367 outside), linkage group 7 used 13,839 SNPs (4920/8919) and linkage group 12 used 8835 SNPs (4343/4492). The remainder of the genome used a subset of exonic SNPs (*n* = 20,115 SNPs). Mean pairwise F_ST_ for all loci tested (*n* = 54,030) ranged between 0.0062 (spring spawners vs. Georges Bank) and 0.0297 (winter spawners vs. Georges Bank). Mean pairwise F_ST_ analysis of each SNP subset tested shows a trend of increased F_ST_ values within some LD blocks as compared to the exonic SNPs on the same linkage group. Mean pairwise F_ST_ for each comparison is shown in Table 8.

**Table 8:** Mean pairwise FST comparisons between sampled populations

	Spring vs. Winter	Spring vs. GB	Winter vs. GB
LG02 in LD	**0.1166**	-0.0189	**0.0981**
LG02 out	0.0025	0.0021	0.0016
LG07 in LD	-0.0062	0.0121	**0.0895**
LG07 out	0.0004	-7.43E-05	0.0001
LG12 in LD	0.0231	0.0766	**0.1726**
LG12 out	0.0022	0.0021	0.0036
Genome	0.0001	-0.0003	0.0003

Mean pairwise F_ST_ calculations for each sampled population show some patterns of divergence between sampled populations, with high mean F_ST_ values between winter spawners and Georges Bank cod in all three LD blocks (shown in bold). LG02 is also highly divergent between spring and winter spawners

We also examined patterns of F_ST_ and LD along each of the chromosomes with linkage blocks. F_ST_ among all three populations was highly correlated with linkage disequilibrium in LG02 and LG12 (Fig. 2, right upper and lower), but was only moderately correlated in LG07 (Fig. 2, right middle). Linkage was highest in LG07, whereas F_ST_ was higher within the linkage groups LG07 and LG12. LG07 also shows a number of chromosomal areas outside the main region of linkage that have occasional high F_ST_, and a low relationship overall between linkage and F_ST_ (Fig. 2, left middle). Variation in the relationship between linkage and F_ST_ suggests other patterns of selection acting on genes on LG07 that would warrant future research.

**Fig. 3:**
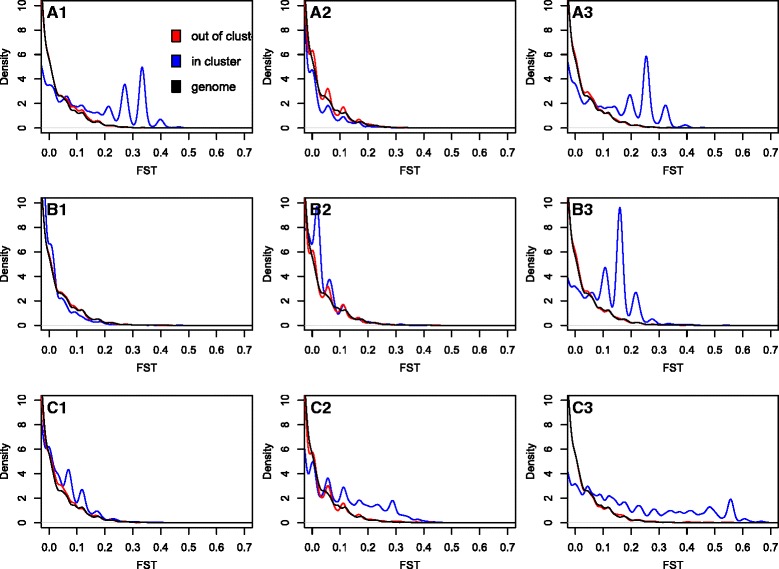
Distributions of pairwise F_ST_ within LD blocks between sampled populations. Pairwise F_ST_ was determined within LD blocks (*blue*), on the same LG but not in the LD block (*red*), as well as the remainder of the genome (*black*) for LD blocks on LG02 (*top row*) LG07 (*middle row*), and LG12 (*bottom row*). *Column 1* is the pairwise FST comparison between spring and winter spawners, *column 2* is spring vs. Georges Bank, and *column 3* is winter vs. Georges Bank

The LD block on linkage group 2 showed a significant increase in higher FST values when comparing spring spawning cod to the winter spawning cod (Fig. 3, A1, mean F_ST_ = 0.1166) as well as between winter spawners and Georges Bank cod (Fig. 3, A3, mean F_ST_ = 0.0981) using a Kolmogorov-Smirnov two-sided test on the F_ST_ distributions (*p* < 1e-10, both comparisons). The LD block on linkage group 7 was also genetically divergent between winter spawners and Georges Bank cod (Fig. 3, B3, mean F_ST_ = 0.0895, KS test *p* < 1e-10). However, the highest differentiation was found between winter spawners and Georges Bank cod within the LD block on LG12 (mean F_ST_ = 0.1726, KS test *p* < 1e-10), with a broader, longer-tailed distribution of F_ST_ values. Mean pairwise F_ST_ values for exonic SNPs on the same linkage groups but not within LD blocks ranged between 0.0001 and 0.0036, and mean F_ST_ values for the remainder of the genome ranged between 0 and 0.0003, which is consistent with one large, outbreeding population.

**Fig. 4:**
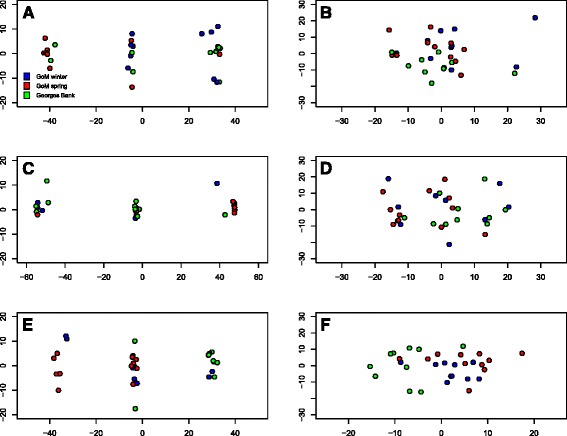
Principal component analyses. PCA was performed on exonic SNPs from LG02 (*top row*), LG07 (*middle row*), and LG12 (*bottom row*). SNPs within the LD block on that LG are shown on the *left*, SNPs outside the LD block on the *right*. In all cases, as expected, SNPs within each LD block separate into three distinct vertical clusters along PC axis 1, indicating the three genotypes that individuals might possess for each LD block. This separation is lost, however, for PCA on SNPs outside the LD block, but in the same linkage group. Note that SNPs outside of the LD block on LG07 (D, above) may signify the presence of another, undiscovered linkage disequilibrium cluster, as the pattern appears diffusely similar to that in C

Principal component analysis was used on SNPs within each LD block, and on SNPs on the same linkage group, but outside the LD block (Fig. 4). In all cases, PCA on SNPs within LD blocks showed three distinct clusters along principal component axis 1, correlating with the three possible genotypes of that LD block (Fig. 4, left). This is in contrast to the remaining exonic SNPs on the same linkage group (Fig. 4, right), where no real clustering is seen between sampled populations, suggesting that all significant divergence is centralized on the three main blocks of linkage disequilibrium.

Finally, we found no evidence for neutral population structure when using the population admixture analysis algorithm NGSadmix. When using k = 2 for SNPs within each linkage disequilibrium block, we found that individual admixture predictions perfectly matched the known genotypes of that individual in the linked loci with the LD block, with homozygous individuals binned into the two populations and heterozygotes having 50/50 admixture, as expected. However, all runs for the entire exonic SNP dataset predicted k = 1 as the most likely population model using the Evanno method of comparing model likelihood scores [45]. All models of k = 2 or higher predicted a high proportion of admixture for all samples and showed no clear differentiation between sampled locations.

## Discussion

In this study, we used whole genome resequencing and single nucleotide polymorphism discovery to describe patterns of genome-wide linkage disequilibrium in the Atlantic cod (*Gadus morhua*) at small spatio-temporal scales within the Gulf of Maine and Georges Bank. Through sequencing and linkage disequilibrium analysis, we found three large clusters of linkage disequilibrium spanning multiple genes, in agreement with the known relative positions of these LD clusters from previously published studies of genomic “islands of divergence” in cod [23, 25–27, 31, 32]. Earlier work had identified such clusters through a set of pedigree-based linkage maps of EST-derived SNPs and microsatellites in controlled crosses. Using custom microarrays, gene lists have been developed to describe the genic content of these linkage regions [32]. Specifically, the LD blocks on LG02 and LG07 have been shown to contain 189 and 297 genes, respectively. Our analysis augments these gene lists to contain 294 and 306 genes, includes an additional 437 genes on LG12, and shows that the extent and genic content of linkage disequilibrium blocks can be detected *de novo* by deep sequencing of wild-caught individuals.

The Atlantic cod genomic islands of divergence are associated with strong natural selection across the Atlantic [25, 26], in the Atlantic Canadian region [27–29], and in the Norwegian Sea [31, 32, 46]. In our samples, large blocks of linkage disequilibrium within LG02, LG07, and LG12 showed a differential pattern of genetic differentiation, with the LD block in LG02 separating between spring and winter spawners within the Gulf of Maine, and the LD blocks within all three linkage groups separating between the winter spawners and Georges Bank cod. This is in direct contrast to SNPs on the same linkage group but outside the LD block, suggesting that the observed differentiation within LD blocks may be due to selection acting on the LD blocks as a unit.

### Temperature and linkage clusters

Local adaptation is not uncommon in marine systems [47]. Studies typically examine local adaptation across large geographic scales, often working at ranges of thousands of kilometers [48–51]. However, evidence is accumulating that highly local adaptation in high gene flow species may be more common than previously thought in both terrestrial and marine systems [52, 53]. Indeed, within Atlantic cod, the genomic region in linkage map LG02 (at ~50 centimorgans) has been shown to have a clinal relationship to average water temperature across the Atlantic, with cod from both Georges Bank and Galway Bay, Ireland (“warmer” locations, mean bottom temp. ~8 °C) largely having one linkage haplotype, and cod from both Newfoundland and Norway (“colder” locations, mean bottom temp. ~0 °C) having the alternate haplotype [26]. We found the same LD block diverging between spring and winter spawning cod within the Gulf of Maine, where near-surface temperatures can vary from 3 °C in late winter to 10 °C in early autumn [54]. Such seasonal variation may facilitate the temporal partitioning of subpopulations of cod if they are adapted to spawning at certain temperature ranges [55].

### Nature of selection in putative ‘supergenes’

Gene located within the LD block of linkage group 2 showed a significant enrichment for genes associated with DNA and chromatin structuring.

This cluster was most divergent between the spring and winter spawning cod within the Gulf of Maine, but was also divergent between winter spawners and Georges Bank. Historically, this linkage disequilibrium block has been shown to differentiate between stationary and migratory cod populations in Norway [31, 32], and have strong allelic associations with seawater temperatures across the Atlantic [26].

It is well understood that temperature plays a large role in the stress response of organisms, including the maintenance of DNA stability [56]. Chromatin plays an important role in the structuring and stability of the DNA molecule, controlling the regulation of gene expression either by relaxing and exposing regions of DNA for easier transcription or by tightening and shutting down transcription. Recently, expression changes in genes involved in chromatin organization and the regulation of DNA replication have been shown to be one of the earliest larval responses to temperature stress in larval zebrafish [57]. In our GO enrichment analysis for LG02, we found several of the major histone proteins (H2A/B, H3a, and H4A), as well as a histone methyltransferase, which methylates the chromatin to alter the ability to transcribe genes, and a histone deacetylase, which removes acetyl groups from chromatin which dampens gene expression. This clustering of histone structural proteins and associated histone methylation and deacetylation genes within the linkage block on LG02 fits the concept of a ‘supergene,’ having several linked loci, inherited as a single unit, with enrichment for related functional genes as a ‘complex phenotype.’

In addition to functional enrichment for histone-related genes, SNPs within the LD block on LG02 are also more likely to be found within exons, as compared to the rest of the genome. 8.9% of the 43,441 SNPs in this linkage block are found in exons, which is a 37% increase over the 6.5% of 2,792,388 SNPs in the rest of the genome. The increase in exonic SNPs, together with a 36% increase in overall SNP density within this LD block, suggests an accumulation and maintenance of genetic variation within this LD block. This is consistent with a balanced, older inversion polymorphism, as time has allowed mutation to accumulate, and selection along with protection from recombination maintains the entire region as a unit, protecting minor deleterious mutations from removal.

While no significant enrichment for GO terms was found in the LD blocks within LG07 or LG12, we know that these genomic regions have been shown to diverge strongly between coastal stationary and offshore migratory cod in Norway [31, 32]. The selection acting on these regions may be acting on one or a few genes of high importance within each LD block, and individuals inherit the region in its entirety due to the repressed recombination of inversion polymorphisms. In our study, we find differentiation between winter spawners and Georges Bank cod in LG12, but no differentiation in LG7 between any of our population samples. This may be due to either our reduced sampling size, or sampling solely from stationary, coastal cod populations.

It is interesting to note that, while these putative supergenes are likely to have been initially protected from recombination by chromosomal inversion [32], the expected result of decreased genetic diversity within inversions is not seen in our data. In fact we see both a larger density of SNPs and a greater proportion of exonic SNPs within the linkage block on LG02. In addition, several unlinked low allele frequency SNPs exist in between the highly linked loci, suggesting that time has allowed mutation to occur within the linkage blocks, as recombination is an unlikely source for the unlinked loci. All of these factors suggest that, if these supergenes arose from chromosomal inversions, they are likely to be older, stable, and adaptive inversion polymorphisms [58, 59].

Caution must be taken in describing potential selection factors within linkage disequilibrium clusters, however, because it is unclear as to whether the entire linkage unit is under selection as a true supergene, or is merely the effect of recent strong selection on one or a few genes with genomic hitchhiking of nearby genes. In the three major LD blocks we described here, the block on LG02 was only one with statistically significant within-cluster enrichment of Gene Ontology (GO) categories, suggesting that the LD blocks on linkage groups 7 and 12 may not have functionally linked genes within them.

### Evidence for population structure

Previous work has shown that genetic differentiation is present within the Gulf of Maine and Georges Bank populations of cod [34, 35]. Most markers in these comparisons showed no structure, but two loci in both data sets showed significant differentiation (the microsatellite ‘Gmo132’ and the *PanI* locus). When Kovach et al. [35] removed these loci from their analysis, no patterns of genetic differentiation were found. Likewise, in our data, both principal component and F_ST_ analyses show that divergence between the sampled populations is limited solely to the linkage blocks within LG 2, 7, and 12, and not throughout the genome. This suggests that some level of adaptive divergence may be responsible for the amount of differentiation historically seen in these genomic regions, though caution must be employed in interpretations of selection in this study due to a low per-population sampling size.

## Conclusions

Strong neutral genetic differentiation between populations implies very low genetic or demographic exchange between them, and as a result, neutral genetic boundaries in fisheries species have long been used to suggest the existence of different stocks [60, 61]. The lack of strong neutral differentiation does not necessarily mean strong gene flow between fish stocks, as large effective population sizes can reduce the effect of differential genetic drift [62]. However, if natural selection generates differentiation, local adaptation and minimal gene flow is often the interpretation. This may not be true, however, as demographic exchange between two populations may remain high [22]. For example, a strong cline in allele frequency at a single locus in the mussel *Mytilus edulis* in southern Long Island Sound is generated by natural selection each summer [63]. However, this genetic divergence does not imply different stocks: the southern population under selection is replenished each spring by strong immigration from the north, and there are few demographic barriers between them. Likewise, allele frequencies of multiple genes in linkage groups LG02, LG07 and LG12 shift over spatial scales of 100 s of miles or temporal scales of decades in cod in Newfoundland [28, 29], implying strong selective changes perhaps due to fishing or environmental shifts. As in the mussel example, such genetic differences do not by themselves imply demographic separation of these populations into separate stocks. They also do not imply that selection is ongoing. The action of strong selection in the past can be visible in current populations if past perturbations of gene flow have not yet been erased by migration.

Previous studies of cod within this system [33] have determined that population structure exists between spawning stocks of cod within the Gulf of Maine. However, these studies relied on markers whose physical locations within the genome were unknown, and often only one or a small number of the markers tested showed genetic divergence [34, 35]. It may be that these markers, which have been interpreted as evidence of population structure, are located within these linkage disequilibrium blocks and are in fact related to their adaptive divergence.

Our data set was designed to provide high coverage of whole genome sequences for relatively small numbers of individuals. Our analysis shows that large regions of select linkage groups appear to be under adaptive divergence between spawning groups. Furthermore, this genetic divergence does not extend to loci on the same linkage map groups within the Gulf of Maine. Together, these results suggest that extensive study of neutral gene divergence within the Gulf of Maine could be accomplished by choosing markers strictly outside known regions of linked polymorphisms. Furthermore, patterns of nucleotide differentiation among linked gene regions might suggest the way natural selection has acted on these regions, and if it continues to shape population genetics of modern cod.

## Additional files

Additional file 1: List of genes found within LD block of LG02. GFF file containing the gene ID, positions, and gene names of the 294 genes found within this linkage disequilibrium block. (TXT 108 kb)

Additional file 2: List of genes found within LD block of LG07. GFF file containing the gene ID, positions, and gene names of the 306 genes found within this linkage disequilibrium block. (TXT 116 kb)

Additional file 3: List of genes found within LD block of LG12. GFF file containing the gene ID, positions, and gene names of the 437 genes found within this linkage disequilibrium block. (TXT 166 kb)

## Abbreviations

cM: Centimorgans
EST: Expressed sequence tag
GB: Georges Bank
GO: Gene ontology
GoM: Gulf of Maine
LD: Linkage disequilibriuml LG, linkage map group
PCA: Principal component analysis
PCR: Polymerase chain reaction
SNP: Single-nucleotide polymorphism
